# Perceptual Rivalry: Reflexes Reveal the Gradual Nature of Visual Awareness

**DOI:** 10.1371/journal.pone.0020910

**Published:** 2011-06-03

**Authors:** Marnix Naber, Stefan Frässle, Wolfgang Einhäuser

**Affiliations:** Department of Neurophysics, Philipps-University Marburg, Marburg, Germany; University of Leuven, Belgium

## Abstract

Rivalry is a common tool to probe visual awareness: a constant physical stimulus evokes multiple, distinct perceptual interpretations (“percepts”) that alternate over time. Percepts are typically described as mutually exclusive, suggesting that a discrete (all-or-none) process underlies changes in visual awareness. Here we follow two strategies to address whether rivalry is an all-or-none process: first, we introduce two reflexes as objective measures of rivalry, pupil dilation and optokinetic nystagmus (OKN); second, we use a continuous input device (analog joystick) to allow observers a gradual subjective report. We find that the “reflexes” reflect the percept rather than the physical stimulus. Both reflexes show a gradual dependence on the time relative to perceptual transitions. Similarly, observers' joystick deflections, which are highly correlated with the reflex measures, indicate gradual transitions. Physically simulating wave-like transitions between percepts suggest piece-meal rivalry (i.e., different regions of space belonging to distinct percepts) as one possible explanation for the gradual transitions. Furthermore, the reflexes show that dominance durations depend on whether or not the percept is actively reported. In addition, reflexes respond to transitions with shorter latencies than the subjective report and show an abundance of short dominance durations. This failure to report fast changes in dominance may result from limited access of introspection to rivalry dynamics. In sum, reflexes reveal that rivalry is a gradual process, rivalry's dynamics is modulated by the required action (response mode), and that rapid transitions in perceptual dominance can slip away from awareness.

## Introduction

While the signals arriving at the human sensory systems typically provide only noisy and ambiguous information about their sources in the real world, introspectively perception seems unified and coherent. Introspection suggests further that at any point in time one can either be aware of a distal item or not, but such awareness cannot be partial. Whether this all-or-none nature of awareness is objectively justified is, however, subject to debate [1], [2], [3] and points to the principled difficulty of relying on introspection (but see [4], [5]).

**Figure 1:**
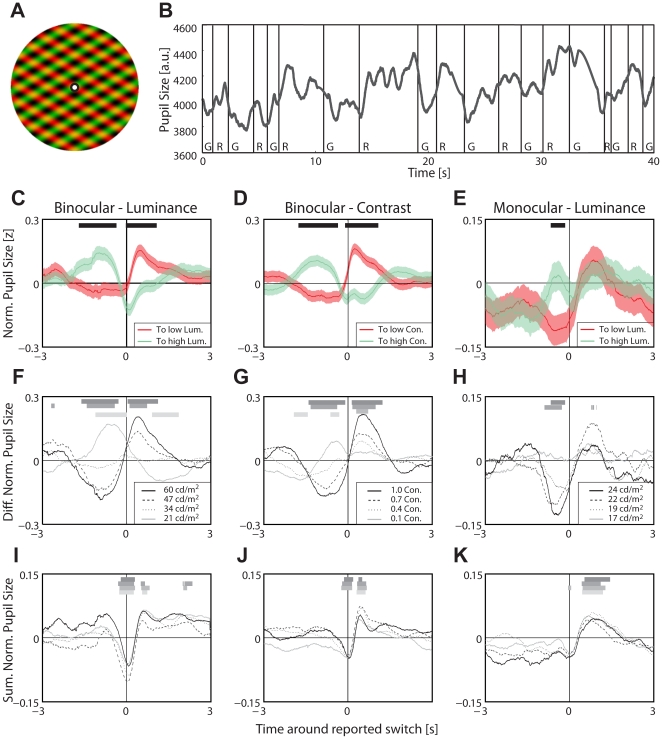
Experiment 1–Pupil size as a function of rivalry dynamics. (**A**) Example of a *monocular* rivalry stimulus presented in experiment 1. Fixate on the center dot to observe rivalry. The perceived luminance of each grating fluctuates over time during which one grating has a more dominant luminance than the other. (**B**) Example of the pupil size as function of time during a single rivalrous trial of one observer. Letters indicate which grating was dominant (R = low luminance red grating, G = high luminance green grating). Black vertical lines are indications of perceptual transitions by the observer. The pupil adapted to the perceived luminance of the dominant percept during rivalry, while physical stimulation was kept constant. (**C–E**) Average pupil size as function of time relative to the perceptual transitions; *red*: transition from high to low luminance or contrast, *green*: transition from low to high luminance or contrast. Light colored patches indicate s.e.m. over all dominance durations. Bars indicate time points were both traces are significantly different at an uncorrected *p*<0.05. (**F–H**) Differences between pupil size traces for the two switch directions as a function of time around transitions for all tested luminance and contrast conditions (legend provides luminance/contrast of changed grating, other remains constant, see Methods), means across observers (n = 8); grey-level coded bars indicate time points at which means are different from 0 at *p*<0.05 . (**I–K**) Sum of pupil size traces, notation otherwise as in panels F–H. (**C–H**) For pupil size as a function of normalized relative time between transitions, see Figure S1.

Studying changes in perception and awareness in the real world presents the challenge that changes in the stimulus (bottom-up signals) interact with changes in their interpretation. To circumvent this bottom-up “confound” we use different variants of rivalry, a phenomenon known for at least a quarter of a millennium [6], [7], [8]. In rivalry, a constant stimulus evokes distinct (usually two) perceptual interpretations (“percepts”). Rivalry occurs when two distinct stimuli, that cannot be fused, are presented to either eye (binocular rivalry, [7]) or when the stimulus itself allows different interpretations, such as the famous Necker cube [9] or the stimulus shown in Figure 1A (monocular rivalry, [6]). To distinguish binocular rivalry from other forms, we here follow the usual convention and subsume the latter as “monocular rivalry” despite simultaneous presentation to both eyes. Rivalry is not restricted to vision, but also observed in touch, audition and olfaction [10], [11], [12].The extent to which all these forms of rivalry exhibit the same phenomenology is subject to debate [13], [14], [15], [16], [17] as is the neural origin of rivalry.

While rivalry is often described as an all-or-none process, a variety of physiological markers show a *gradual* modulation around the time of transition between two percepts: eye-position [18], [19], [20], (micro-)saccade frequency [20], [21], pupil dilation [22], [23], blink frequency [20], or gamma-band activity [24], [25]. As with awareness in general, however, this conflict between discrete phenomenology and continuous physiology, may be a consequence of relying on introspection and subjective report. Hence we here combine rivalry with objective measures to probe visual awareness without relying solely on introspection.

Numerous studies have attempted to pinpoint neural processes underlying rivalry. Conceptually, local and modality specific processes have often been put forward: the representations of each percept mutually inhibit each other and loss of dominance follows from neuronal adaptation (“fatigue”) of the dominant percept [26], [27], [28]. In addition, evaluative higher-order processes could account for the reversals between percepts as these trigger changes in visual awareness by reorganization of activity (e.g., [29]). Several studies support a low-level account of rivalry, linking transitions between percepts to spatial activity patterns in V1 [30], [31], [32] or indicating correlations between fluctuations of early visual activity and changes in visual awareness [31], [33], [34], [35]. Other studies, however, have shown that higher brain areas are linked to awareness [29], [36], [37], [38]. To reach consensus on the subject, several authors have argued that rivalry may occur at different levels of the visual hierarchy [39], [40], [41], [42], [43], [44]. Supporting this proposition, Haynes and Rees [45] show that different forms of rivalry (in their case between eyes or between colors) have representations at distinct locations in the visual processing stream. It seems thus likely that both high and low level mechanisms contribute to initiation of rivalry [44].

One possible reason for the lack of consensus on the neuronal origin of rivalry and awareness is the effect of attentional and motor processes that add an additional difficulty to the interpretation of imaging data. For example, attention could selectively activate or modulate distinct regions; subjectively attending a specific feature, such as one orientation in two overlapping gratings with different orientations, activates neurons processing that feature in early visual areas [46]. Similar to attending one feature, the allocation of attention to one percept could result in the activation of neurons processing the features tied to the corresponding percept. The activated brain regions during rivalry could thus be a consequence of attentional processes rather than the actual process involved with visual awareness. Attentional mechanisms are indeed known to affect brain regions differently during rivalry [32]. Regions responsible for the allocation of attention to salient events [47] seem to be involved with spontaneous perceptual switches as well [36], [48], [49], [50]. Also, decreasing attentional resources to a rivalrous stimulus slows the rate of alternations in rivalry [51], [52], and selectively attending a percept prolongs its dominance [16], [53]. In summary, attentional processes may strongly affect rivalry and interact with the neural representations of the rivaling stimuli.

A key issue that bedevils many studies on rivalry is their reliance on introspection (i.e., subjective report). Although occasional physical changes in the stimulus can verify that observers try to achieve a veridical report (“catch trials”), four fundamental issues persist: First, the motor-act of reporting itself may affect perception [54], [55], [56]; second, the report mode might restrict response possibilities (e.g., button presses allowing only discrete reports); third, very brief dominance periods of one percept might not suffice to trigger a report; and forth, catch trials might not mimic the entire phenomenology of rivalry. Here we use three strategies to overcome these issues: first, we use two reflexes–pupil response and optokinetic nystagmus–as objective indicators of percept; second, we test different input devices, a discrete (button press) and a continuous (joystick) one; third we simulate discrete and gradual transitions in catch (“simulated”) conditions.

While changes in pupil size are typically thought of as a reflex to changes in illumination, there are many studies showing cognitive effects on pupil size [e.g., 57,58,59,60,61,62,63,64,65,66]. In the context of rivalry, transitions induce pupil dilation [22], [23] and the pupil light reflex is diminished during suppressed periods [67], [68], [69], [70]. Here we find that if stimuli of different luminance or contrast are presented to either eye, pupil size follows the percept rather than the physical stimulus allowing us to use pupil size as one objective indicator of rivalry.

When observers are presented a large coherently moving field (such as when looking outside from a moving train), their eyes usually show an optokinetic nystagmus (OKN, [71]): slow phases try to match the stimulus speed to keep the retinal image stable and are interrupted by fast phases that reset the eye in orbit. Here we employ OKN slow phase speed as second objective measure of rivalry. In the present context, this measure is of particular interest, as previous studies have described both rivalry and the resulting OKN as all-or-nothing mechanisms [72], [73], [74], [75], [76]. Combining OKN with an analog input device allows us to challenge this interpretation. Using both pupil size and OKN allows us to verify that results are general and not restricted to specific methods of measurement or stimuli.

Using OKN and pupil size, using discrete and continuous response modes, and using sharp and piece-meal simulated transitions, we address three questions: is awareness temporally “all-or-none” or gradual, are the results explainable by piece-mealing, and do reflexes have access to different levels of processing than introspection?

## Results

### Experiment 1–Pupil size as measure of rivalry

To robustly induce rivalry, ten observers were presented two stationary gratings that were distinct in color (red/green) and orientation (+60° or −60° relative to the vertical). In a *monocular* rivalry condition [6], both gratings were overlaid as plaid and presented to both eyes simultaneously (Figure 1A; note that we stick to the term *monocular* rivalry although the stimulus was presented to both eyes); in *binocular* rivalry, each grating was separately presented to one eye through a stereoscope. In addition, either the green grating's luminance (binocular and monocular condition) or contrast (binocular only) was varied across 4 levels between experimental trials, while the red grating was kept identical at an intermediate level of luminance or contrast. During each 5-minute trial, both gratings were constant, and observers were asked to indicate by pressing a button, which grating they perceived. Despite a constant stimulus, pupil dilation depended on the luminance of the dominant percept (Figure 1B): if the grating of higher luminance or contrast was perceived dominant, the pupil size was smaller than if the other grating was dominant, and significantly so for binocular luminance (t(9) = 6.79, *p*<0.001) and for binocular contrast (t(9) = 4.99, *p*<0.001) conditions. During monocular rivalry, pupil size was significantly smaller during the second half of a dominance duration (i.e., close to the next perceptual transition) if the dominant percept was brighter than the suppressed percept (t(9) = 2.34, *p*<0.05). We align pupil traces to the times when observers reported transitions. The average over these aligned traces shows that the difference in pupil dilation is largest just before and after the transition (Figure 1C–E) and levels off after about 1.5s. Some of the leveling off in the average trace may be attributed to the high variability of time to the subsequent transition (i.e., the high variability in dominance durations). We control this confound by performing the analysis in a normalized time frame: we resample the time between each pair of subsequent perceptual transitions (i.e., a dominance duration) to a single fixed length before averaging. This time-normalized representation still shows a gradual change in pupil from one transition to the next transition (Figure S1). This rules out dominance-duration variability as sole source of the graduality of transitions. Importantly, the difference in pupil size between percepts increases with increasingly distinct stimuli, while the time course remains rather similar (Figure 1F–H). This pupil response is therefore also distinct from a generic biphasic pupil response associated with the perceptual transition as such, which is independent of the polarity of the transition (low to high or high to low) and degree of dissimilarity between the rivaling stimuli (Figure 1I–K, cf. [22], [23]). In sum, experiment 1 demonstrates that pupil size–both in monocular and binocular rivalry-follows the perceived rather than the physical stimulus. A similar result has recently been found independently and was first reported as abstract [77] together with a presentation of the current results [78]. The most important aspect is that pupil dilation seems to indicate a *gradual* transition between the two perceptual states (Figure 1B). Whether these gradual transitions are truly a property of rivalry rather than of the pupillary response itself, shall be addressed in experiment 2.

### Experiment 2–Graduality: a true property of rivalry

Experiment 1 leaves open whether the time course of the pupil around transitions reflects a gradual nature of rivalry or just sluggish pupil dynamics. To distinguish these two alternatives, we simulate abrupt rivalry transitions and compare the speed of pupil size changes around these simulated transitions to real rivalry transitions.

**Figure 2:**
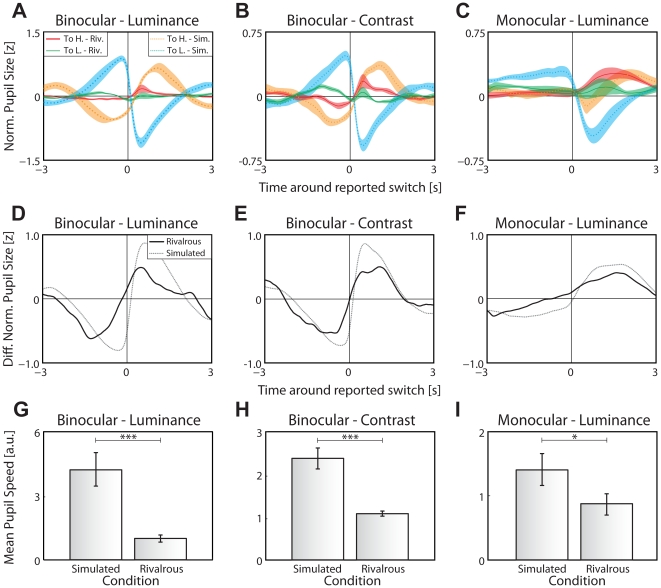
Experiment 2–Pupil graduality. (**A–C**) Average pupil size as a function of time relative to the perceptual transitions for rivalry (green and red traces) and physical stimulus changes (simulated rivalry, orange and cyan traces) per condition. (**D–F**) Difference in average pupil size per for rivalrous transitions (black solid traces) and simulated transitions. To compare rivalrous and simulated transitions, average pupil traces were normalized by dividing through the distance between the maximum and minimum of each trace per observer and trial. (**G–I**) Average horizontal pupil speed in a 0.2 time window around the pupil speed's zero crossing, mean and s.e.m. across observers for simulated (*left*) and real (*right*) rivalry. Pupil size was more gradual during rivalrous transitions as compared to simulated transitions.

All methodological aspects of experiment 2 were identical to that of experiment 1 with the following exceptions: First, rather than using multiple contrast or luminance levels, we only used the stimulus conditions for which the difference in luminance or contrast between the rivaling stimuli had been maximal in experiment 1; second, we simulated rivalry in half of the trials by switching presentation of the two gratings per dominance duration (“simulated rivalry”). Dominance durations in these trials were based on the preceding rivalrous trial and perceptual transitions consisted of abrupt switches between images of both “rivaling” gratings. Visual inspection of the average traces indicates that pupil size changes more abruptly during simulated transitions (Figure 2A–C, dotted traces) than during rivalrous transitions (Figure 2A–C, solid traces) for all conditions. This is particularly evident in the difference plot between the two transition polarities (Figure 2D–F), where effects of the transition itself (i.e., irrespective of polarity) are subtracted out. Although this is already suggestive of rivalrous transitions being more gradual than simulated transitions, such apparently faster transitions in the *average* trace of simulated trials, may in principle, still be a consequence of decreased variance (jitter) between the times of actual transitions (simulated or real) and their report. We controlled for this potential confound by calculating the steepness (speed of transition) in a 0.2 second window around each change in pupil size tied to a perceptual transition. When using the z-normalized pupil size for computing pupil speeds, pupil speeds around simulated transitions were significantly larger than around rivalrous transitions for all conditions (Binocular luminance: simulated: 4.4±1.2, rivalrous: 0.9±0.5, t(7) = 7.73, *p*<0.001; Binocular contrast: simulated: 2.5±0.8, rivalrous: 1.2±0.3., t(7) = 6.53, *p*<0.001; Monocular luminance: simulated: 1.5±0.5, rivalrous: 0.8±0.5, t(7) = 2.95, *p*<0.05; Figure 2G-I, all values in units of z-normalized pupil size divided by 200 ms). Finally, to exclude artifacts of trial-wise normalization, we computed the same analysis for unnormalized pupil sizes and find the same pattern (t(7) = 5.95, *p*<0.001; t(7) = 5.02, *p*<0.01; t(7) = 3.53, *p*<0.01, respectively). These data confirm that transitions in simulated rivalry are indeed significantly steeper than in real rivalry. Hence, the gradual nature of a change in dominance is not a mere consequence of sluggish pupillary dynamics, but it is a true property of perceptual transitions in rivalry.

### Experiment 3–Generality of graduality and limitations of report

Pupil size indicates the gradual nature of rivalry. To address whether this phenomenon is restricted to the effect of perception on pupil dynamics or a general property of rivalry, we use an alternative objective measure. Especially in the context of binocular rivalry and moving stimuli, the velocity of the slow phase component of the optokinetic nystagmus (OKN) provides such an alternative.

**Figure 3:**
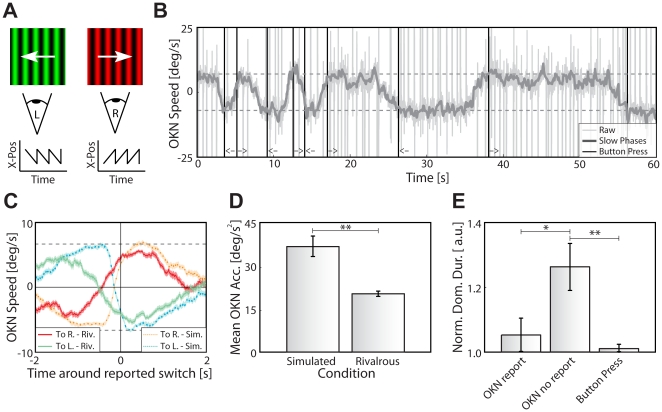
Experiment 3–OKN as a function of rivalry dynamics. (**A**) Example of *binocular* rivalry stimuli and OKN patterns. Rivalry and OKN were induced by presenting dissimilar gratings with opposite movement directions to each eye separately. (**B**) Example of the derivative of the horizontal eye position (OKN speed) as function of time during a single trial of one observer (dark grey). OKN's fast phases (light gray spikes) were removed and interpolated, and the resulting trace was smoothed. Black vertical lines indicate perceptual transitions; arrows denote movement direction of the dominant percept. Dashed grey horizontal lines at (-)6.67 deg/s indicate speed of the rivaling stimuli. The OKN speed of slow phases gradually increased and decreased as a function of time to transition. (**C**) Average OKN speed for rivalry transitions (green and red traces) and physical stimulus changes (simulated rivalry, orange and cyan traces), mean and s.e.m. across all dominance durations. OKN speed was more gradual during rivalry transitions as compared to physical changes of stimulus direction. (**D**) Average horizontal eye acceleration in a 0.2 time window around the OKN speed's zero crossing, mean and s.e.m. across observers for simulated (*left*) and real (*right*) rivalry. Acceleration during a perceptual transition, an indication of graduality of the OKN speed signal, was lower for rivalrous trials. (**E**) Normalized dominance durations per report conditions, mean and s.e.m. across observers. Dominance durations were normalized by dividing by the median dominance duration per observer. OKN-based dominance durations (default parameter settings) differ significantly in a condition when observers in addition report dominance by button press (*left bar*) as compared to trials without active report (*middle bar*). In the parameter regime, OKN-based dominance durations in the report condition do not differ from those based on subjective (button press) reports (*right bar*).

Eight observers were presented a grating moving to the right to one eye and a grating moving to the left to the other eye, both at a speed of 6.7 deg/s (Figure 3A). Our analysis is based on the horizontal eye velocity during the slow phases of the OKN, whose direction significantly depends on whether the leftward or rightward moving grating is perceived as dominant (horizontal velocity left: −1.15±0.27 deg/s, right: 1.09±0.20 deg/s, t(7) = 5.49, *p*<0.001; Figure 3B). Note that the low gain of about 0.16 ( = 1.1/6.7) is a consequence of averaging over the whole period, including times around the transitions: Similar to the pupil signal in experiment 1, the OKN velocity changed smoothly around a perceptual transition (Figure 3C, solid traces) and leveled off to baseline around 2 s afterwards. Similar to experiment 2, we excluded the possibility that the smoothness of the transition is a property of the OKN velocity rather than of the rivalry process. We simulated rivalry by interleaving experimental trials in which both gratings for each eye drifted in the same direction and physically switched their direction with the same temporal statistics as the preceding rivalrous trial (i.e., the same randomized dominance durations). In these cases, transitions in OKN velocity are more abrupt and take less time to follow the change in stimulus direction (Figure 3C, dotted traces). The difference in the steepness of transition is quantified by the average eye acceleration during the transition, which is significantly larger for simulated than for real perceptual transitions (simulated: 36.8±3.7 deg/s^2^, rivalrous: 21.7±1.6 deg/s^2^, t(7) = 4.74, *p*<0.01; Figure 3D). In line with the pupil data from experiment 2, the gradual nature of transitions indicated by the OKN is therefore a true and general property of rivalry. This raises the question as to whether rivalry is governed by a continuous process that is shadowed by a discrete (binary) response mode.

**Figure 4:**
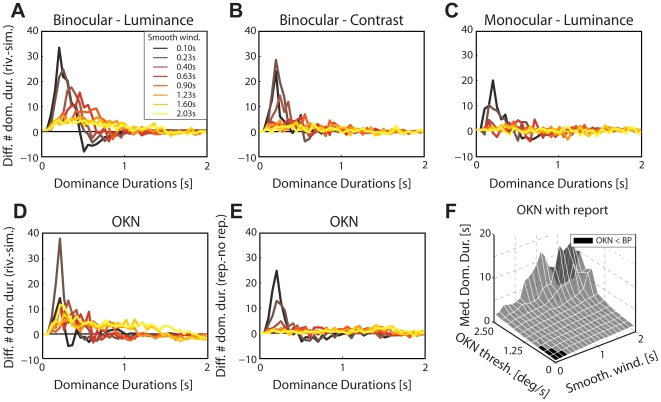
Parameter settings and dominance durations. (**A–D**) Difference between dominance-duration histograms for real-rivalry and simulated-rivalry based on pupil data (experiment 2, panels A–C) or OKN data (experiment 3, panel D) for different widths of the smoothing window applied to the raw trace. For each parameter setting, histograms were computed separately for dominance durations in real rivalry and simulated rivalry with the same binning (25ms bins). Histograms were then subtracted bin-wise; positive values imply that the respective bin (i.e., dominance duration) contained more incidences in the rivalrous condition as compared to the simulated condition, and negative values imply the converse. For short smoothing windows an abundance of short dominance durations is observed in real rivalry. This suggests that there exist short dominance durations in real rivalry that go unreported by the observers (see text). (**E**) Difference between dominance-duration histograms between report (active) and no-report (passive) trials based on OKN (experiment 3) and their dependence on the smoothing window. Histograms were subtracted bin-wise (25 ms bins); positive values indicate that the respective dominance-duration bin contains more incidences in the report condition than the no-report condition. (**F**) Median OKN-based dominance duration as a function of smoothing window and OKN sign change threshold. Black patches indicate when OKN-based dominance durations were significantly shorter than button-press-based dominance durations. In real-rivalry trials there is no ground truth as to whether transitions-based on pupil and OKN-are veridically identified. However, for a wide range of smoothing parameters there is only a small or no difference in such pupil and OKN-based dominance durations and those obtained from the button presses. For short time windows on the smoothing filter of the reflexive measurements and low thresholds, however, we observe significantly shorter dominance durations based on reflexes than based on button presses for rivalrous and reported trials as compared to simulated and unreported trials, respectively. This possibly reflects the difficulty for short dominance durations to be reported (see text). These data leave us confident that pupil and OKN sign changes are a reliable indicator of transitions in rivalry and may provide access to short dominance durations that observers cannot consciously report.

If fluctuations in visual awareness of percepts are in fact governed by a gradual process, why do its measurements suggest an all-or-none nature [79], [80]? One plausible hypothesis sees the typically discrete response mode (button press) responsible. There are two ways button presses can influence measured dominance durations. First, button-press report may miss very brief dominance durations [81]; second, the act of overtly reporting *per se* may influence perception. For the first issue, it is conceivable that short dominance durations are not reported, because they do not reach awareness or an internal integration criterion for report. To test this, we use pupil size and OKN-slow-phase velocities and their sign changes (i.e., a directional change in pupil size and crossing of the zero-velocity line) as objective indicator of perceptual dominance. While in real rivalry it remains open for principled reasons whether very brief reflex-based dominance durations are noise (i.e., false alarms for transitions by reflexes) or true dominance periods missed by overt report, simulated rivalry provides such ground truth. Since simulated rivalry is based on real-rivalry button-press data, noise related to the reflex itself would show up as transitions in both conditions, while truly missed short periods of dominance would only be present in real rivalry. In general, pupil-based and especially OKN-based dominance durations are largely consistent with button-press-based dominance durations; for some regime of parameter settings, however, there is an abundance of short dominance durations in the signals for real rivalry as compared to simulated rivalry (Figure 4A–D). In addition, latencies between physical transitions and button presses are longer (pooled pupil conditions: 0.53s±0.09s; OKN: 0.66s±0.03s) than between physical transitions and reflexive sign changes (pupil: 0.41s±0.10s, t(23) = 3.62, *p*<0.01; OKN: 0.42s±0.05s; t(7) = 5.08, *p*<0.001). Thus, it is likely that observers are unaware of or fail to report short dominance durations because of these latencies. Furthermore, information on intermediate states of rivalry [81], [82] can also be lost when relying solely on button-press data. The second option, a direct effect of overt report on dominance durations is also supported by our data. For parameters that closely match OKN-based to button-based dominance durations, OKN-based dominance durations are shorter if observers actively report their percept than if they merely watch the stimulus passively (t(7) = 3.21, *p*<0.05; Figure 3E; Figure 4E–F). This result stresses the effect of report on dominance durations and is in line with earlier observations [54], [56]. Note that we did not measure no-report conditions for pupil experiments, because pilot data had already indicated that the pupil is less reliable in determining perceptual dominance as compared to OKN, which is confirmed by the data we report here (∼65% overlap of report-based dominance with pupil-based dominance versus ∼90% overlap with OKN-based dominance, [78]). In sum, reflexes show effects of discrete overt report on rivalry: first, a possible miss of brief dominance durations; second, a direct effect of report on perception. Given this strong impact of report, it is likely that the commonly used discrete response mode (button presses) may shadow the gradual nature of rivalry.

### Experiment 4–Continuous input device and piece-meal rivalry

**Figure 5:**
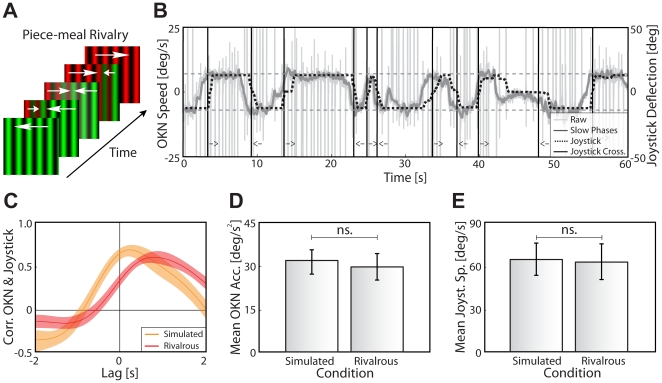
Experiment 4–Piece-mealing and joystick report. (**A**) Example of piece-meal rivalry. Piece-meal rivalry consists of gradual transitions between percepts during which parts of both percepts are spatially intermixed. (**B**) OKN speed (notation as in Figure 2B) compared to joystick deflection (*dashed black line*) for a single rivalrous trial of a single observer. Joystick deflection was a good indicator of rivalry dynamics and delineated intermediate states. (**C**) Cross-correlation of OKN speed and joystick deflection (positive lags: OKN leading), mean and s.e.m over dominance durations; *red:* real rivalry, *orange:* simulated rivalry (piece-meal). Observers had longer latencies and lower peak correlations for rivalrous trials as compared to simulated rivalry trials. (**D**) OKN acceleration in a 0.2s time window around OKN zero-crossings, mean and s.e.m. across observers for simulated (*left*) and real rivalry (*right*). Unlike for the abrupt changes in experiment 2, OKN acceleration was not different from real rivalry trials for piece-meal simulation. (**E**) Joystick speed in a 0.2s time window around zero crossings of joystick position, mean and s.e.m. across observers for simulated (*left*) and real (*right*) rivalry. Speed of the joystick during a perceptual transition did not differ across conditions.

To directly test whether the subjective report has at least some access to the gradual process underlying rivalry, we asked the same eight observers to report their perceptual state, that is the “amount by which one percept is dominant”, by a continuous input device (an analog joystick) while being presented the same rivalrous stimulus as in experiment 3. To mimic the perceptual impression more closely, the simulated condition here implemented a wave-like transition rather than the abrupt change of experiment 3 (Figure 5A; details see Materials and Methods). The subjective report through the joystick was very consistent with the objective OKN measure (Figure 5B), as reflected in the high peak cross-correlation between joystick deflection and OKN slow-phase velocity (simulated: r = 0.81s±0.03, rivalrous: r = 0.70s±0.03; Figure 5C). Observers even indicate intermediate states in which both percepts dominate equally by centering the joystick for a prolonged period (e.g., Figure 5B at around 15s and 45s). The joystick response lagged, however, considerably behind the OKN response, which is reflected in the time of peak in the cross correlation (simulated: 0.51s±0.10s, rivalrous: 0.86s±0.09s) as well as in the time between zero crossings of OKN velocity and joystick (simulated: 0.54s±0.07s, rivalrous: 0.84s±0.09s). This suggests that very brief dominance periods, which are potentially reflected in the reflexes, either do not reach awareness or fail to be reported. This could partially account for the difference between subjective and objective measures described above (Figure 3E; Figure 4E–F). Consistent with the interpretation of a lag between a low-level transition in percept and its availability to introspection, simulated transitions exhibit a significantly shorter lag between OKN and joystick than real transitions (peak cross-correlation: t(7) = 3.96, *p*<0.01; time between OKN and joystick crossing: t(7) = 2.54, *p*<0.05). Similarly, the peak correlation between OKN and joystick is significantly larger for simulated rivalry (t(15) = 2.30, *p*<0.05; separated by switch direction). Unlike in experiment 3, the OKN transitions themselves were comparable between simulated and rivalrous trials (simulated: 31.8±4.6 deg/s^2^, rivalrous: 29.7±4.5 deg/s^2^, t(7) = 1.07, *p*>0.25; Figure 5D) as were the speed characteristics of the joystick (simulated: 65.2±9.6 deg/s, rivalrous: 63.3±9.7 deg/s, t(7) = 0.64, *p*>0.50; Figure 5E). Joystick speed and OKN mean acceleration in a 0.2s time window around transitions were also correlated (mean r = 0.65±0.07, *p*<0.001). As such, there was no difference between simulated and real rivalry in the response measures, indicating that the simulation of the transition between percepts captured the perceptually relevant aspects of the real rivalry transition. Despite possible misses of rapid changes in awareness as shown in experiment 3, experiment 4 demonstrates that the gradual nature of rivalry transitions revealed by the objective measures (reflexes) grossly corresponds to the subjective percept. Hence, the seeming all-or-none nature is likely to be an artifact of the response mode. Interestingly, a wavelike transition between percepts offers a possible explanation for a considerable amount of the observed graduality. If this hypothesis holds true, this would suggest that rivalry is globally gradual, but local differences in perceptual dominance contribute to its graduality. Consequently, visual awareness would be gradual in time because of fragmentation in space. Irrespective of whether piecemeal rivalry is the cause, our data show graduality of rivalry in time, whose accessibility is influenced by response mode.

## Discussion

The time course of two reflexes–pupil dilation and OKN–points to a continuous process underlying rivalry. Subjective perception has access to this continuous process, though this access is substantially delayed and in turn influences rivalry dynamics. Our results have implications not only for current models of rivalry, but also for the debate as to whether visual awareness is globally all-or-none or a gradual phenomenon (e.g., [1], [2]).

Remarkably, OKN and pupil dilation, which are typically thought of as reflexive behaviors to physical changes, depend on the percept rather than on the stimulus. These “reflexes” serve as rapid measures of the perceptual state and thus as an objective indicator of a seemingly subjective state: the awareness of either perceptual interpretation. Previous studies showed that the pupillary reflex is suppressed if contrast increments are presented in the suppressed percept [67], [68], [69], [70]. We show, however, that the pupil size is continuously modulated as a function of dominance between incongruent luminance and contrast stimuli. We show that pupil size can be used as novel method to objectively measure both *binocular* and *monocular* rivalry. A recent study has suggested that *monocular* and *binocular* rivalry share common underlying mechanisms [13]. In the present study, the increased pupil size modulation for binocular rivalry as compared to monocular rivalry could be a consequence of different luminance settings or different perceived luminance. It is, however, possible that both rivalry types generally differ in rivalry strength or exclusivity of dominance [17]. Such differences could be related to the fact that during monocular rivalry only the patterns (color, luminance and orientation) rival, while patterns *and* eyes rival during binocular rivalry. Despite the quantitative difference in pupil size modulation, its robust occurrence in both rivalry types suggests that monocular and binocular rivalry share a similar mechanism driving pupil size.

We have discovered several additional advantages of the utilization of reflexes during rivalry. Reflexes have a shorter latency than subjective report, and they avoid an influence on perception by the response mode itself. The influence of report on rivalry is consistent with the observation that report mode affects dominance durations [54], [56]. Here we show that rivalry is slowed down when observers do not report the percept. Attentional factors may–at least in part–account for this effect as they have been found to strongly relate to rivalry dynamics. In particular, distracting observers with a dual-task during rivalry, slows down the rate of alternations [51], [52]. This result elegantly concurs to our finding that passive viewing of rivalry similarly slows down the rate of alternations. In agreement with the idea that attention is responsible for alternation rates in rivalry, a recent study demonstrated that the cortical thickness of the superior parietal lobe (SPL), a brain area anatomically close to parietal areas that get activated during both shifts in attention [83], [84] and perceptual transitions in rivalry [36], [85], relates to individual differences in alternation rates in rivalry [86]. Furthermore, perceptual transitions are generally initiated at the most salient [87], [88] or attended location [89]. As such, our data suggest that deployment of attentional resources to the stimulus through active report of perception, increases the likelihood of transitions between interpretations of a stimulus, and thus increases switch rates during rivalry.

The notion of rivalry as an “all-or-none” process probably dates back as early as Necker [9] who described the transitions of his bi-stable cube as “sudden and involuntary” (p. 336). However, for the case of binocular rivalry, Wheatstone [7] observed that “When complex pictures are employed in the stereoscope, various parts of them alternate differently” (§14). Our results imply that such fractionation of percepts (piece-meal rivalry, also see [90]) may–at least in part–be responsible for the gradual transitions indicated by the reflexes. Recent evidence similarly indicated that fluctuations in the visual awareness of percepts during binocular rivalry are gradual because sensitivity to probes presented in the suppressed percept slowly rises as a function of time towards the next transition, and vice versa, sensitivity to probes in the dominant percept slowly declines over time as a perceptual transition gets more likely to occur [3]. In one experiment, these authors exclude piece-meal periods from analysis based on observer report. In the light of our present results, the reliability of such introspection-based analysis is questionable. Specifically, latencies in responses (and awareness) might prevent observers from reporting piece-meal periods veridically. In addition, piece-meal rivalry can manifest itself either as discrete transitions or as different levels of perceptual fading, which challenges the dissociation between an all-or-none experience of rivalry and a gradual underlying adaptation process. Both effects can lead to a globally gradual impression of rivalry, although the former retains discreteness locally. In any case, our data show that rivalry is globally gradual over time and that piece-meal rivalry offers *one* possible explanation that is consistent with the data. We have chosen a sinusoidal simulation of piece-meal rivalry to incorporate graduality of changes in dominance (for details see Methods). We observe similar joystick reports in simulated and real rivalry with these settings, which indicates that the simulation was realistic. Nonetheless, studying which simulation parameters reflect the most “natural” perceptual transitions and how they affect the reflexes could quantify the contribution of piece-mealing to graduality further and remain interesting issues for future research.

Neuronal explanations of rivalry generally describe that distinct pools of neurons encode percepts separately and inhibit each other reciprocally (e.g., [91], [92], [93]). Adaptation of neurons in the dominant pool eventually leads to a decrease in inhibition and thus to a transition in dominance towards the previously inhibited and rivaling pool. Our data do not directly contradict this notion or constrain the role of inhibitory connections, but they point in the direction that spatiotemporal adaptation, a process also subject to interocular grouping (e.g., [94], [95]), may play a role in the graduality of rivalry dynamics. The fractionation of percepts has been linked to representations in early visual areas because the size of fractions during piece-meal rivalry changed with eccentricity congruently with the human cortical magnification in these areas [96]. This relation with cortical magnification in early visual areas has been argued to be at least partially responsible for visual field anisotropies in visual awareness in a variety of phenomena [97]. Recent studies also demonstrated that brain activity in V1 is highly correlated with transitions in visual awareness [30], [31], [32]. It is thus not unlikely that the gradual nature of visual awareness has its roots in spatiotemporal mechanisms controlled by early visual areas. Even if the loci of rivalry representations would be known, a different question remains as to which processes are responsible for the (gradual) transition as such. One study argues that high-level fronto-parietal areas produce activity related to the initiation of perceptual transitions [36] but does not find evidence for whether this activity precedes and thus triggers changes in awareness. A recent study does find a causal relationship between parietal areas and perceptual alternations by disrupting activity with transcranial magnetic stimulation and measuring alternation rates during rivalry [86]. It is still, however, uncertain whether the application of TMS to parietal areas disrupts the ability to report perceptual transitions or disrupts the actual initiation of an alternation. Nonetheless, the initiation of changes in awareness by higher-order areas could be well reflected in the observed pupil dilation around a transition, and may have its roots in processes related to decision making [22], [65], motor planning [23], or attention allocation [58]. On the other hand, activity in later areas might be a net result of activity fluctuations related to dominance in rivalry processed by low-level visual areas. Indeed, we find that–the presumably low-level controlled–reflexes have much shorter latencies than high-level subjective report as a response to perceptual transitions. We now can–with the use of reflexes–objectively examine whether high-level effects are a result of or a cause for changes in awareness during rivalry [29].

### Conclusions

Reflexes reveal that rivalry is a gradual process, its dynamics are affected by the response mode, and fast changes in dominance can slip away unnoticed (or unreported) by observers. Consequently, reflexes allow access to earlier (subconscious) levels of perception, which are unavailable to awareness, and thus stress the limits of relying on introspection alone.

## Materials and Methods

### Observers

Ten observers (age: 19–48, seven female, three male) participated in experiment 1, eight observers (age: 19–48, six female, two male) in experiment 2, and another group of eight observers (age: 19–28, five female, three male) in experiment 3 and 4. All had normal or corrected-to-normal vision, were naïve to the purpose of the studies, and gave informed written consent before each experiment. The experiments conformed to National Guidelines for psychological experiments as laid down in the Ethics Regulations of the German Psychological Society and to the ethical principles laid down in the Declaration of Helsinki.

### Apparatus

*Binocular* stimuli were presented on 21 inch Samsung Syncmaster CRT screens at a viewing distance of 30 cm. *Monocular* stimuli were presented on a 21 inch EIZO Flexscan CRT screen at a viewing distance of 70 cm. The display refresh rate of all screens was 85 Hz and the resolution was either 1152×864 pixels (experiment 1 and 2) or 1280×1024 (experiments 3 and 4). Screens were gamma corrected, achieving a linear mapping of pixel values to stimulus luminance. Each colored grating used a single gun of the monitor, whose CIE color space coordinates (x,y) were (0.623, 0.344), (0.287, 0.609), and (0.151, 0.065) for the red, green, and blue gun, respectively. Stimuli were generated on an Optiplex 755 DELL computer, using Matlab (Mathworks, Natick, MA) and the Psychophysics toolbox [98], [99] and EyeLink toolbox [100] extensions. *Binocular* stimuli were dichoptically presented with two monitors by projecting them with a mirror stereoscope to each eye separately. Mirrors were transparent for infra-red light (i.e., cold mirrors), allowing an infrared sensitive camera (EyeLink 2000, SR Research, Osgoode, ON, Canada), positioned behind the mirrors, to track both pupil sizes and direction of gaze at a rate of 500 Hz. *Monocular* stimuli were presented with a single screen and observers looked through a transparent mirror that reflected infrared light (i.e., a hot mirror) from the eyes to the eye-tracking camera (Eyelink “Tower Mount”) that recorded at 1000 Hz. In all experiments, the observers' head was supported by a chin- and forehead-rest. In all pupil experiments, observers fixated a fixation dot (0.8° diameter) centered over the stimulus. The eye-tracker was (re)calibrated before each experiment and after each break (see procedure).

### Stimuli and Procedure–Experiment 1

Three different stimulus sets were used in four separate sub-experiments, conducted on separate days. The first stimulus set consisted of sinusoidal gratings with a spatial frequency of 2 cycles per degree and opposing orientations (i.e., −60° and +60°), one grating colored red and the other green (Figure 1A). The red grating's luminance ranged from 0.011 cd/m^2^ to 18.5 cd/m^2^. The green grating's luminance varied across trials (21.1, 33.5, 47.0, or 60.4 cd/m^2^). Binocular rivalry was induced by presenting both red and green grating separately to each eye. Gratings were presented in a gray (33.5 cd/m^2^) annulus of 10 degree diameter. The whole stimulus was framed by a 2° wide rectangular bar consisting of a high spatial frequency noise pattern, to ensure a steady binocular fusion of the backgrounds. Stimulus' orientation, color, and presentation to the corresponding eyes were randomized during each experiment.

The second stimulus set similarly consisted of sinusoidal gratings but instead of luminance, contrast was varied across trials. The red grating had a Michelson contrast of 0.6 with a maximum luminance of 11.2 cd/m^2^. The green grating's contrast was varied, having either one of four Michelson contrasts of 0.1, 0.4, 0.7, or 1.0, corresponding to a minimum-maximum luminance of 30.2–36.9, 20.1–47.0, 10.1–57.0, or 0–67.1 cd/m^2^, respectively.

The third stimulus set consisted of overlapping sinusoidal gratings with a spatial frequency of 1 cycle per degree. These stimuli induced a form of *monocular* rivalry during which the visibility of each grating fluctuated over time. These fluctuations consisted of one grating being more visible (i.e., a higher perceived luminance) than the other until its perceived luminance decreased while the other grating's luminance increased. Similar to the first stimulus set, luminance of the green grating was varied across trials. The red grating had maximum luminance of 5.7 cd/m^2^. The green grating's luminance was varied across 4 levels, 16.9 cd/m^2^, 19.4 cd/m^2^, 21.8 cd/m^2^, or 24.2 cd/m^2^. In contrast to the first and second stimulus set, the overlapping gratings were presented monoptically without the mirror stereoscope.

Each of the four possible grating pairs was presented four times per sub-experiment, resulting in a total of 16 trials (4×4). A single trial lasted 120 seconds and was followed by a 20 second break. During each trial, observers viewed the stimuli and indicated the dominant percept with two buttons. As dominance of each percept fluctuated, intermediate states occurred in which both percepts were equally dominant. Nonetheless, observers were instructed to always indicate one (i.e., the most dominant) percept as dominant. After 8 trials, observers were allowed to take a 5 minute break.

### Stimuli and Procedure–Experiment 2

In experiment 2, all stimulus aspects were identical to experiment 1, except that only the stimuli, for which the green grating was most distinct from the red grating, were used: binocular green grating luminance of 60.4 cd/m^2^, binocular green grating contrast of 1.0, and monocular green grating luminance of 24.2 cd/m^2^. For each of the 3 conditions (binocular luminance, binocular contrast, monocular), observers conducted 4 trials of 3 minutes each. Two of these trials were real-rivalry trials as in experiment 1, two were simulated-rivarly trials, in which alternations in dominance were simulated by switching the presentation of each stimulus (i.e., the red or green grating) per dominance duration. Distribution of dominance durations in each simulated-rivalry trial was based on the report of the preceding real-rivalry trial.

### Stimuli and Procedure–Experiments 3 and 4

In experiment 3 and 4, a large (height: 50.6 deg, width: 37.1 deg) drifting sinusoidal grating (0.15 cycles/deg) was presented to each eye, one dark and red (peak luminance 17.4 cd/m^2^), the other light and green (68.2 cd/m^2^) on a dark background (<0.01 cd/m^2^). Both gratings were moving in opposite lateral directions (left and right) at a speed of 6.7 deg/s.

In experiment 3, we simulated rivalry by presenting the same stimulus to both eyes and physically switched colors and drifting directions simultaneously. Statistics of dominance durations were matched to the preceding real rivalrous trial. In experiment 4, the perceptual transition was simulated as a smooth transition through a state of piece-meal rivalry. The previously invisible stimulus became visible by spatially moving it over the dominant stimulus from one side to the other. During a simulated transition, complementary parts of both stimuli were thus visible simultaneously but divided by a vertical border at which both stimuli transparently overlapped (width: 14 degrees). The transitions followed a sinusoidal movement pattern with half periods (i.e., dominance duration) of either a fraction of 0.8 or 1.2 of the median dominance duration from the preceding rivalrous trial.

Experiments 3 and 4 consisted of 4 conditions, which differed in presentation condition (real rivalrous versus simulated rivalry) and response mode (active versus passive) and were repeated twice (8 trials per experiment). In active report, observers were either instructed to indicate the dominant percept by holding down the arrow key on a keyboard (experiment 3) or by deflecting a joystick (experiment 4). They were told to release buttons or keep the joystick in a middle position when no percept was dominant. During passive trials, observers were instructed to just watch the stimulus. Trials lasted 5 minutes and were followed by short breaks.

### Analysis

In all binocular experiments the pupil sizes of both eyes were highly correlated (r>0.99 in all cases) as was the OKN in experiment 3 and 4 (r>0.98), such that only the right eye was used for analysis in all cases. Pupil size was based on its diameter as recorded by the Eyelink tracker system. Eye-position and pupil size were interpolated with a cubic spline fit during periods in which observers blinked their eyes. For analyzing how the pupil size developed around a perceptual transition (Figure 1C–K), pupil size was normalized to z-scores per trials (i.e., trial mean subtracted and divided by standard deviation). Horizontal eye velocity as function of time is given by the derivative of the eye's raw horizontal position. To obtain the slow phase of the OKN, multiple thresholds were applied to the absolute values of convolution filtered (square smoothing window of 0.1s width) velocity traces (>.5 deg/s), acceleration (>.1 deg/s^2^), and the derivative of acceleration (>.01 deg/s^3^) of the horizontal eye position signal to remove all fast phases from the OKN speed trace (Figure 3B, slow phases). The zero crossing points of the OKN's slow phase component velocity were assumed to be objectively defined perceptual transitions. The obtained OKN zero crossings points were filtered for random noise in OKN speed. If crossing points were preceded by a relatively low maximum or minimum (i.e., those extremes were between a "sign-change threshold" of 1.6 or −1.6 deg/s), they were assumed to be a result of intermediate percepts and removed from the analysis of transition speeds. Mean velocity of the OKN was calculated per slow phase and smoothed using a sliding square window of 0.1 seconds width. Dominance durations were calculated from either the button presses, joystick deflection baseline crossings, or from distance between the unfiltered (i.e., no removals because of low preceding extremes) zero crossing points of the OKN. For rivalrous trials, the latency of responses of both the button presses and joystick deflections as compared to the OKN were based on the median time between a button press or joystick crossing and the preceding OKN crossing that indicated perceptual transitions. For simulated trials, the latency of the button presses, joystick crossing, pupil or OKN sign changes were based on the median time from a simulated transition. If the latency was longer than 2 seconds, it was assumed to be a missed transition and was therefore removed from analysis.

## Supporting Information

Figure S1 **Pupil size during rivalry in a normalized time frame.** **(A–C)** Normalized mean and transparent s.e.m. pupil size (z-score) as a function of relative time between perceptual transitions per dominant percept for each stimulus set. The time axis was normalized to unit length between transitions by re-sampling all pupil traces per dominance duration (3000 samples) before averaging (details see [3], [18]). For the red trace the transition from high luminance to low luminance percept thus happens at time 0 and back at time 1, while the reverse holds for the green trace. In this periodic time frame time, 1 for the red trace corresponds to time 0 for the green trace and vice versa. The pupil increased or decreased in size when the dominant percept was low or high in luminance or contrast, respectively. **(D–F)** Mean differences in pupil size traces between the percepts as a function of relative time between perceptual transitions. Grey values of traces indicate the level of luminance or contrast of one of the gratings (the other rivaling grating had a fixed intermediate level of luminance or contrast). The degree of pupil size modulation to the luminance or contrast of the dominant percept depended on the difference in luminance or contrast between the rivaling percepts. Thick patches at the top indicate when traces are significantly (*p*<0.05) different from each other (panels A–C) or from 0 (panels D–F).
(TIF)
