# Supplementary figures and images for: Perceptual Rivalry: Reflexes Reveal the Gradual Nature of Visual Awareness
Source: PLoS One. 2011 Jun 3;6(6):e20910. doi: 10.1371/journal.pone.0020910 (PMC3109001; doi:10.1371/journal.pone.0020910)

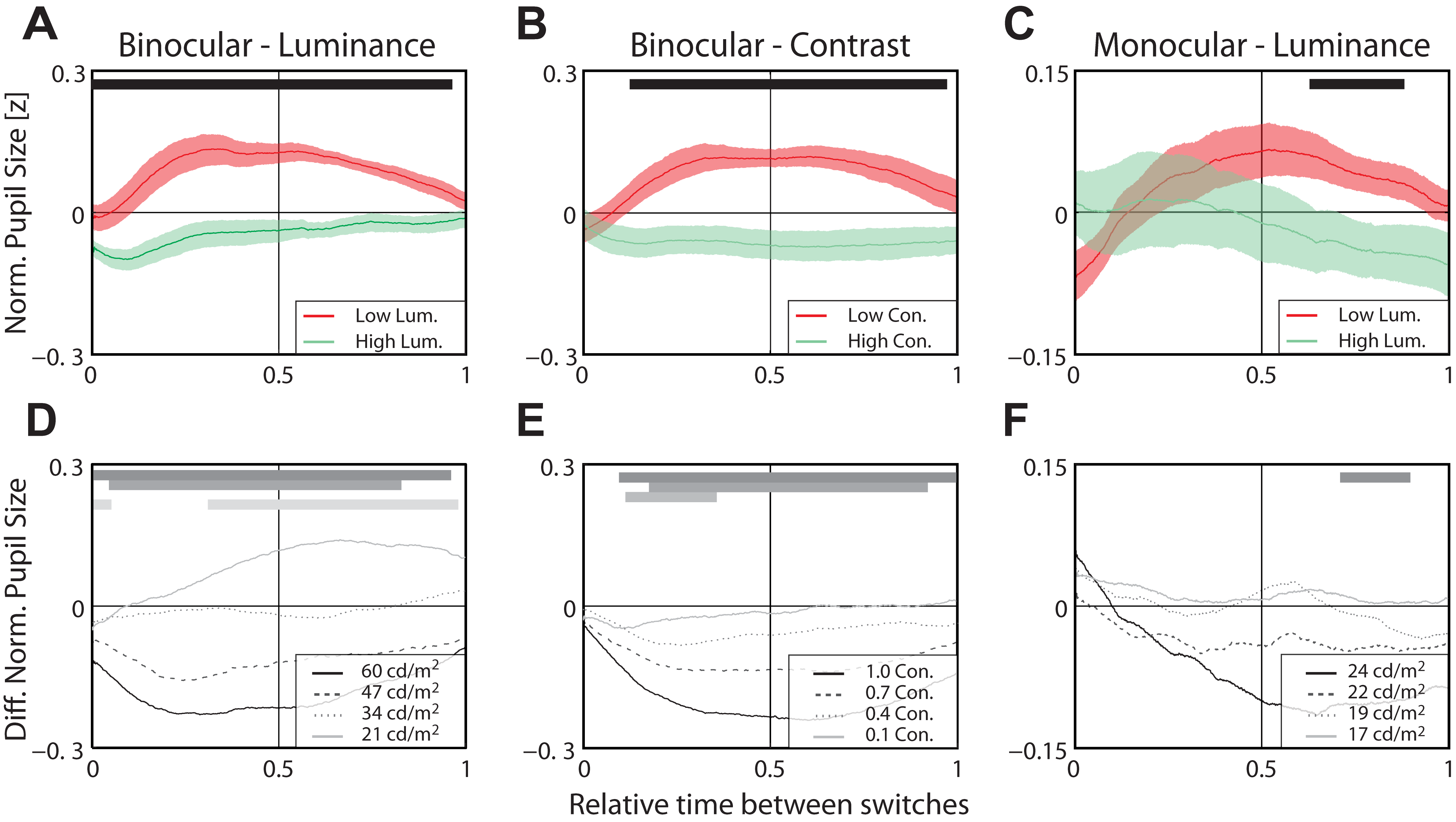

Supplement: Figure S1 — Pupil size during rivalry in a normalized time frame. (A–C) Normalized mean and transparent s.e.m. pupil size (z-score) as a function of relative time between perceptual transitions per dominant percept for each stimulus set. The time axis was normalized to unit length between transitions by re-sampling all pupil traces per dominance duration (3000 samples) before averaging (details see [3], [18]). For the red trace the transition from high luminance to low luminance percept thus happens at time 0 and back at time 1, while the reverse holds for the green trace. In this periodic time frame time, 1 for the red trace corresponds to time 0 for the green trace and vice versa. The pupil increased or decreased in size when the dominant percept was low or high in luminance or contrast, respectively. (D–F) Mean differences in pupil size traces between the percepts as a function of relative time between perceptual transitions. Grey values of traces indicate the level of luminance or contrast of one of the gratings (the other rivaling grating had a fixed intermediate level of luminance or contrast). The degree of pupil size modulation to the luminance or contrast of the dominant percept depended on the difference in luminance or contrast between the rivaling percepts. Thick patches at the top indicate when traces are significantly (p<0.05) different from each other (panels A–C) or from 0 (panels D–F). (TIF) [file pone.0020910.s001.tif]
